# Preventative Effects of Vitamin E on Testicular Damage and Sperm Parameters in the First-Generation Mice Pups due to Pre- and Postnatal Mancozeb Exposure

**DOI:** 10.1155/2019/4763684

**Published:** 2019-08-01

**Authors:** Esmaeil Saddein, Tahereh Haghpanah, Seyed Noreddin Nematollahi-Mahani, Fatemeh Seyedi, Massood Ezzatabadipour

**Affiliations:** ^1^Anatomical Sciences Department, School of Medicine, Kerman University of Medical Sciences, Kerman, Iran; ^2^Physiology Research Center, Neuropharmacology Institute, Kerman University of Medical Sciences, Kerman, Iran; ^3^Department of Anatomy, Jiroft School of Medicine, Jiroft University of Medical Sciences, Jiroft, Iran

## Abstract

The present study aimed to evaluate the effects of vitamin E on mancozeb-induced testis damage of the first-generation pups during intrauterine and lactating periods. Two groups of pregnant NMRI mice received 500 mg/kg mancozeb (MNZ) as MNZ group and 200 mg/kg vitamin E as MNZ+vit.E group before receiving MNZ. In addition, a vehicle and a control group were designed every other day in gestation and lactation periods. The male pups from each group were maintained until adulthood (8-10 W). The left testes and epididymides were removed following the sacrifice of the pups. Then, they were weighed, and sperm parameters including number, viability, motility, and morphology and testis structure were evaluated. A significant decrease occurred in sperm parameters of the mancozeb-treated pups compared to the control and vehicle groups. Treatment with vitamin E reversed the deleterious effects of MNZ to a nearly normal condition. Testis parameters including the weight, gonadosomatic index, seminiferous tubule diameters, and Johnsen's score, as well as the number of germ cells such as spermatogonia, spermatocyte, spermatid, and Sertoli, decreased significantly in the MNZ group, compared to the amount in the control and vehicle groups. Interestingly, the treatment with vitamin E was reversed in most of these parameters. Based on the results, the exposure of pups to mancozeb during pregnancy and lactating periods negatively affects the reproductive system of male pups. However, the coadministration of vitamin E could prevent the deleterious effects of mancozeb on sperm and testis parameters.

## 1. Introduction

Mancozeb, as a pesticide approved by the American EPA (Environmental Protection Agency) [1] with minimal toxicity in mammals [2], is increasingly used around the world [3, 4]. The production of mancozeb may continue by 2020 at least due to low price and global demand. However, some researchers believe that the adverse effects of mancozeb-contaminated agriculture products and water have been increasing since the first appearance of mancozeb in 1948 [1]. In this regard, neurotoxic effects and Parkinson's like symptoms [5], fetal brain development dysregulation [6], and thyroid hormone disruption are highlighted, especially among women [7]. In addition, antispermatogenic and antiandrogenic effects of mancozeb on the adult male reproductive system were already reported [4]. Spermatogenesis disruption was reported after administering high dose of mancozeb on the adult male mice and rabbits [8, 9]. However, there are some gaps in the data and unresolved questions regarding the impact of mancozeb on some developing organs such as reproductive systems [10] which was suggested in a review paper [1].

Some reports found a correlation between mancozeb administration and oxidative stress, along with some evidence of mancozeb-induced disruption of hypothalamus-pituitary-gonadal axis [4, 11]. Overproduction of reactive oxygen species (ROS) is known as a risk factor for male fertility [12, 13]. Vitamins are famous as strong antioxidants. In the present study, vitamin E (alpha tocopherol), as a liposoluble antioxidant [14] with their high impact on fertility potential [15, 16], was used to evaluate its impact on mancozeb-induced damage of male reproductive system development. Further, the effects of long-term exposure of embryos and neonate mice to mancozeb and the preventative effects of vitamin E on sperm parameter disturbance and testicular damage in the first-generation mice pups (f1) were evaluated.

## 2. Material and Methods

### 2.1. Chemicals

The materials were purchased from Sigma Company (MO, St. Louis, USA). In addition, Mancozeb (manganese-zinc ethylene bis(dithiocarbamate)) with 80% purity, wettable powder (CAS: 8018-01-7) was obtained from Indofil chemical company, India.

### 2.2. Animals

NMRI mice were purchased from Pasteur Institute of Iran and kept in the standard condition of animal house such as the temperature of 22 ± 1°C and a 12:12 light/dark photoperiod (6am, light on). Further, the mice were fed by a standard hard-pelleted (Pasteur Institute, Iran) diet and had free access to tap water. Furthermore, they were housed in corncob bedding in mouse cages. The study protocol was approved by the animal ethics committee of Kerman University of Medical Sciences* (ethical code: ir.kmu.rec.2014.137).*

### 2.3. Experimental Design

In this study, a total of 18 adult female mice, aged 8-10 weeks, and six 10-12-week-old adult male mice were used for mating. Three female and one male mouse were caged overnight and every next morning, the formation of vaginal plug was checked. The day after vaginal plug observation was considered as gestational day 1. The pregnant mice were randomly divided into four groups (n=4 each) including control (first), vehicle (second), and two mancozeb-treated groups (third & fourth). The first group received no treatment while the second group received 0.1 ml/10 gr body weight (BW) olive oil. Further, the animals in the third group (MNZ) received 500 mg/kg BW (1/10 LD50) mancozeb dissolved in olive oil [17] and the fourth group (vit.E+MNZ) received vitamin E (with ≥ 95.5% purity, Sigma Aldrich, CAS: 10191-41-0, semisynthetic acetate ester of *α*-tocopherol) 200 mg/kg, dissolved in olive oil and mancozeb. The animals in the latter group received vitamin E thirty minutes before mancozeb. This interval time was selected based on the two facts: (1) the duration of vitamin E absorption through the small intestine; (2) the duration of reaching to its proper level in plasma after gavage [18]. Except the control group, the animals received different treatments including olive oil, mancozeb, and vitamin E by gavage every two days based on the study design. The treatment was initiated on the second day of pregnancy and continued until the end of lactation period so that the embryos and offsprings could be exposed to mancozeb and vitamin E through the placenta [19] and blood-milk barrier [20, 21]. A total of 116 pups were born from 16 mothers (n=4); 59 of them were male (18, 17, 11, and 13, respectively, belonging to control, vehicle, MNZ, and vit.E+MNZ). After weaning, the first-generation pups (randomly selected 10 pups from each group) were kept on a standard diet until adulthood (8-10 weeks). In addition, the adult male pups were weighed and sacrificed by cervical dislocation. Finally, the testes and epididymis were removed and weighed and accordingly the sperm and testis parameters were assessed.

### 2.4. Evaluation of Sperm Parameters

Sperm parameters including number, viability, motility, and morphology were evaluated based on the WHO guideline (5th edition, 2010). The left cauda epididymis was put in 1 ml prewarmed Ham's F10 medium supplemented with BSA (15 mg/ ml), cut with surgical scissors, and incubated for 20-25 minutes at 37°C and CO_2_ 5% in the air for evaluating the sperm parameters.

### 2.5. Sperm Number

An equal volume of sperm suspension was mixed with 10% formaldehyde. Then, 10 *μ*l of the diluted solution was placed on an improved neubauer hemocytometer and observed by an optical microscope (Olympus BX 51, Tokyo, Japan) at ×400 magnification.

### 2.6. Sperm Viability

Sperm viability was assessed by using Eosin-Nigrosin staining. Then, 10 *μ*l sperm suspension was mixed with 10 *μ*l Eosin-Nigrosin stain, and a smear was prepared on a clean glass slide after two minutes. At least 200 sperms were evaluated by an optical microscope (Olympus BX51, Tokyo, Japan) at ×400 magnification. Finally, the dead and live sperms were observed dark-red and pale-pink, respectively.

### 2.7. Sperm Motility

First, 10 *μ*l of the sperm suspension was put on a clean glass slide, covered with a 22×22 mm coverslip. The sperms were observed by an optical microscope (Olympus BX51, Tokyo, Japan) at ×400 magnification and the motility of at least 200 sperms was assessed in 5 fields and was recorded as the percentage of motile sperms. Finally, the sperm motility was classified as progressively motile, nonprogressive motile, and nonmotile.

### 2.8. Evaluation of Sperm Morphology

The analysis of sperm morphology was performed on Eosin-Nigrosin stained slides by an optical microscope (Olympus BX51, Tokyo, Japan) at ×200 magnification. At least 200 spermatozoa were assessed in each slide and the percentage of any type of abnormality in the head, neck, and tail was recorded.

### 2.9. Measurement of Gonadosomatic Index (GSI)

Before cervical dislocation, the animals in each group were weighed by a digital balance. In addition, the testis weight was determined after surgery. Testis to body weight ratio was calculated and recorded as the percentage of GSI [22].

### 2.10. Assessment of Testicular Structure

The samples included 28 testes from four groups (10/each group). The tissues were fixed in 10% formaldehyde, dehydrated with ascending grades of ethanol (70-90-100%), and embedded in paraffin, and 5 *μ*m sections were prepared by using a rotary microtome (Leitz, Germany). The slides were stained with hematoxylin and eosin (H&E) and studied under a light microscope (Olympus BX51, Tokyo, Japan). Johnsen's score (spermatogenesis) was used to report the changes in the seminiferous epithelium quality [23, 24] on a 10-point scale as follows: complete spermatogenesis and normally organized tubules (10), many spermatozoa but germinal epithelium disorganized (9), only a few spermatozoa available in the section (8), no spermatozoa but many spermatids (7), only a few spermatids (6), no spermatozoa or spermatids, but many spermatocytes (5), only a few spermatocytes (4), only spermatogonia (3), no germ cell, but only Sertoli cells (2), and no germinal and Sertoli cells (1).

### 2.11. Quantitative and Qualitative Assessment of the Seminiferous Tubule Parameters

First, 20 seminiferous tubules per testes from seven mice were randomly selected in each experimental group, and the parameters such as the diameter of tubules and lumens, thickness of germinal epithelium, number of germinal epithelium cells (spermatogonia, spermatocyte and spermatid), and Sertoli cells were measured [25]. Finally, the qualitative parameters of seminiferous tubules such as the degenerative changes of spermatogenic cells, disorganization of tubules, detached epithelium, and sloughed cells were evaluated.

### 2.12. Statistical Analysis

First, Kolmogorov-Smirnov test was used to determine the normal distribution of variables. Then, ANOVA test was used, along with Tukey test as a post hoc when the distribution of variables was normal. Otherwise, Kruskal-Wallis test was used. The results were reported as mean ± standard error of mean (SEM). The difference was statistically significant when p <0.05.

## 3. Results

During pregnancy, no obvious effect was found for maternal mancozeb intake such as clinical symptoms, mortality, and abortion.

### 3.1. Evaluation of Epididymal Sperm Number and Viability

**Figure 1:**
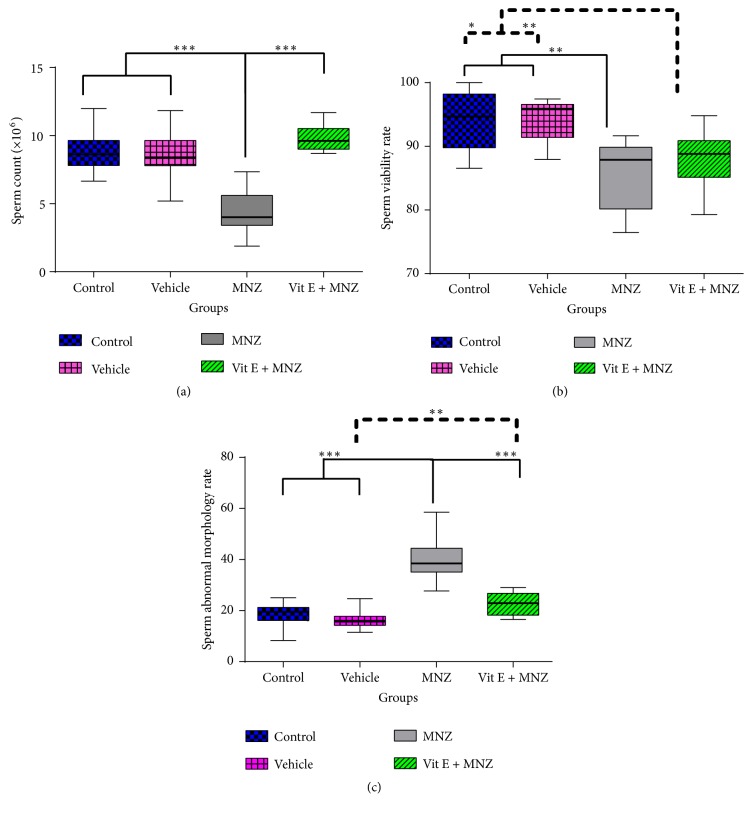
The effect of mancozeb administration alone and with vitamin E during intrauterine life and lactating periods on sperm count (a), viability (b), and abnormal morphology (c) in the different groups (n=10). MNZ: mancozeb. **∗**, *∗∗*, and *∗∗∗* indicate p<0.05, p<0.01, and p<0.001, respectively.

A significant decrease was observed in the epididymal sperm number and viability in the mancozeb-treated mice, compared to the control and vehicle groups (Figures 1(a) and 1(b)). Supplementation with vitamin E significantly increased the number of sperms, compared to the mancozeb-treated group (p<0.001). Sperm viability was comparable between the MNZ and vit.E+MNZ groups but significantly lower than sperm viability in the control and vehicle groups.

### 3.2. Evaluation of Sperm Morphology

**Figure 2:**
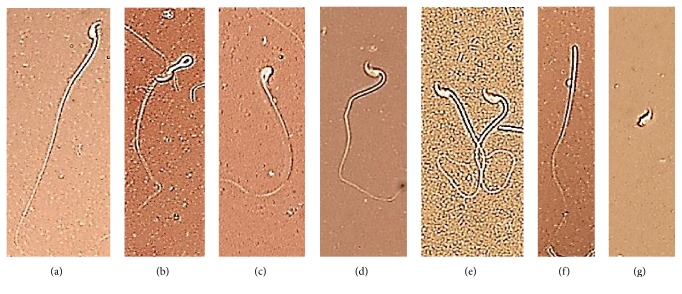
Normal and abnormal sperm forms: (a) normal, (b) looped midpiece, (c) pin head, (d) bend neck, (e) coiled tail, (f) headless, and (g) tailless (Eosin-Nigrosin staining, magnification: ×*640*).

As displayed in Figure 1(c), mancozeb administration led to a sharp increase in the abnormal morphology of sperms, compared to the amount in other groups (p<0.001). As shown in Figure 2, the abnormal forms of sperm morphology such as pin head, looped midpiece, bent neck, coiled tail, headless, and tailless (Figure 2) were abundant in the MNZ group than the amounts in the other groups. Interestingly, supplementation with vitamin E improved the sperm morphology significantly, compared to the MNZ (p<0.001) and vehicle groups (p<0.01).

### 3.3. Evaluation of Sperm Motility

**Figure 3:**
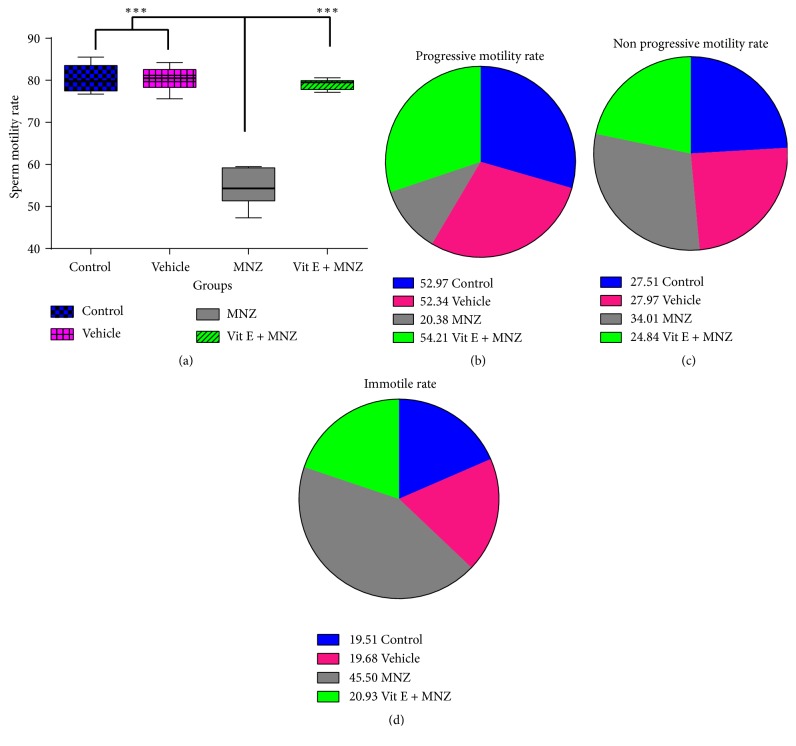
The effect of mancozeb administration alone and with vitamin E during intrauterine life and lactating periods on sperm motility (a) and progression (b–d) in the different groups (n=10). The percentage of progressive motile sperm was lowest level in MNZ group, whereas this value in vit.E+MNZ group was near to those of control and vehicle groups (b). The immotile sperm rate was highest in MNZ group (d). MNZ: mancozeb. *∗∗∗* indicates p<0.001.

Figure 3 illustrates sperm motility and progression in different groups. The lowest rate of motile sperms was detected in the MNZ group, which was significantly lower (p<0.001) than that of the other groups, while the administration of vitamin E increased the value significantly, compared to the MNZ group (p<0.001). The highest rate of progressive sperm motility was observed in the vitamin E plus mancozeb group, compared to the MNZ group (p<0.001), vehicle (p<0.01), and control (p<0.05) groups (Figures 3(b), 3(c), and 3(d)).

### 3.4. Evaluation of Body Weight, Testicular Weight, and Gonadosomatic Index (GSI)

**Figure 4:**
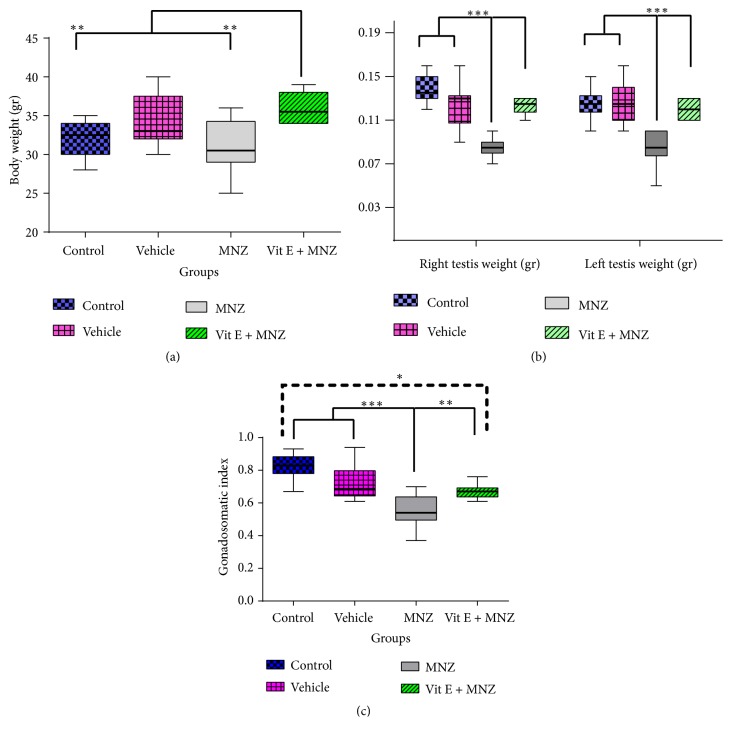
The effect of mancozeb administration alone and with vitamin E during intrauterine life and lactating periods on the weight of body (a) and right and left testis (b) and gonadosomatic index (GSI) (c) in the different groups (n=10). MNZ: mancozeb. *∗*, *∗∗*, and *∗∗∗* indicate p<0.05, p<0.01, and p<0.001, respectively.

Figure 4 displays body weight, testicular weight, and GSI ratio in the f1 offsprings. As shown, body weight remained fixed statistically in the different groups, while it increased significantly in the vit.E+MNZ group compared to the control and MNZ groups (Figure 4(a)). In addition, the testis weight in the MNZ group decreased significantly (p<0.001), compared to the weight in the control and vehicle groups, while it increased significantly (p<0.001) in the vit.E+MNZ group, compared to the MNZ group (Figure 4(b)). Further, GSI of the MNZ group decreased significantly (p<0.001), compared to the control and vehicle groups, while it increased significantly (p<0.05) in the vit.E+MNZ group, compared to the MNZ group (Figure 4(c)).

### 3.5. Evaluation of Testis Structure

**Figure 5:**
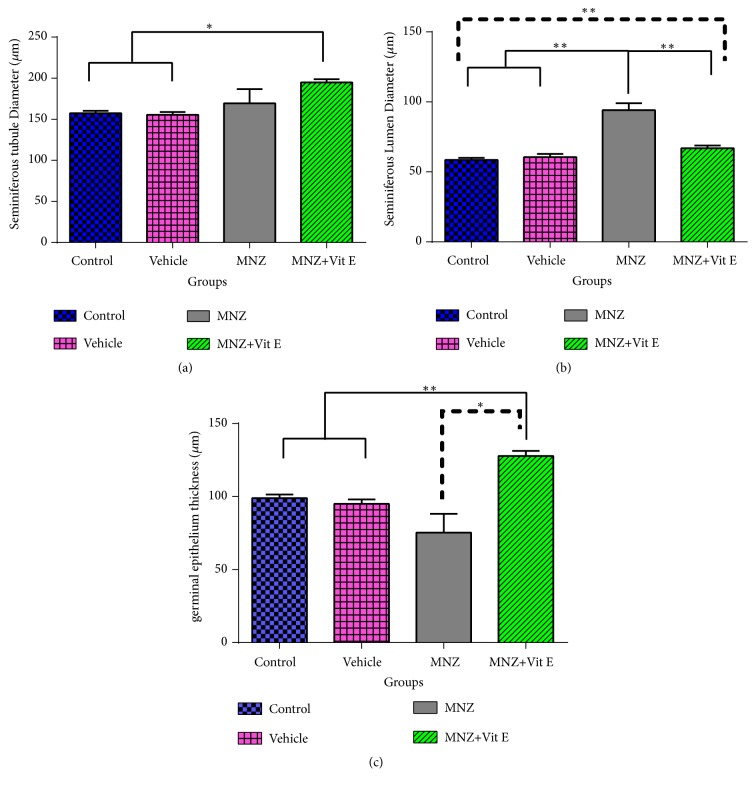
The effect of mancozeb administration alone and with vitamin E during intrauterine life and lactating periods on testis structure; seminiferous tubule diameter (a), seminiferous lumen diameter (b), and germinal epithelium thickness (c) in the different groups (n=7). MNZ: mancozeb. **∗** and *∗∗* indicate p<0.05 and p<0.01, respectively.

As illustrated in Figure 5, no significant difference was observed in the diameter of seminiferous tubules among the control, vehicle, and MNZ groups, while it increased significantly (p<0.05) in the vit.E+MNZ pups, compared to the control and vehicle groups (Figure 5(a)). In addition, treatment with mancozeb increased the diameter of seminiferous lumen significantly, compared to the control and vehicle groups (p<0.01), while supplementation with vitamin E decreased the lumen diameter significantly (p<0.01) compared to the MNZ group. This diameter in the vit.E+MNZ group was still significantly higher than that of the control group (Figure 5(b)). Further, a significant decrease occurred in the diameter of germinal epithelium in the MNZ group, compared to the control and vehicle groups. However, it was significantly higher in the vit.E+MNZ group than that of the control (p<0.01), vehicle (p<0.01), and MNZ (p<0.05) groups (Figure 5(c)).

### 3.6. Evaluation of Spermatogenic Cell Line

**Figure 6:**
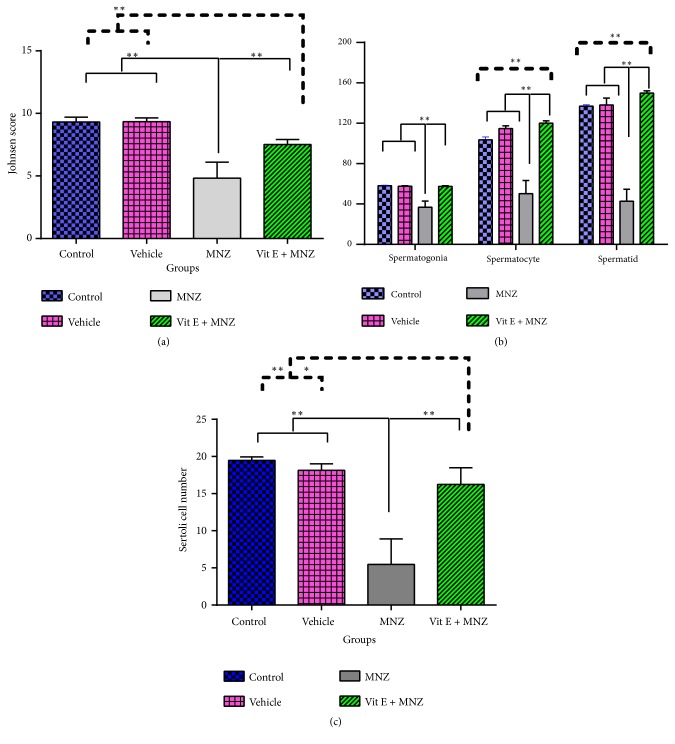
The effect of mancozeb administration alone and with vitamin E during intrauterine life and lactating periods on the Johnsen score (a) and number of spermatogenesis cell lines (b); spermatogonia, spermatocyte, spermatid, and also the number of Sertoli cells (c) in the different groups (n=7). MNZ: mancozeb. **∗** and *∗∗* indicate p<0.05 and p<0.01, respectively.

Figure 6 displays Johnsen's score and the changes in spermatogenic cell line in the different groups, respectively. A significant decrease was observed in the MNZ group (p<0.01) compared to the control and vehicle groups. Although the administration of vitamin E increased Johnsen's score significantly compared to the MNZ group (p<0.01), it failed to reach a significant level compared to the control and vehicle groups (p<0.01) (Figure 6(a)).

The mean number of spermatogenic cell lines (spermatogonia, spermatocyte, and spermatid cells) in the vehicle group was approximately similar to that of the control group (p>0.05). In addition, a significant decrease in the number of spermatogenic cell lines and Sertoli cells was detected in the MNZ group (p<0.01), compared to the number in the control and vehicle groups. However, administering vitamin E increased the number of the spermatogenic cells line significantly, compared to the number in the MNZ group (p<0.01) (Figure 6(b)). Furthermore, a significant increase occurred in the number of spermatocyte and spermatid cells in the vit.E+MNZ group, compared to that of the control animals (p<0.01) (Figure 6(b)). However, pretreatment with vitamin E increased the number of Sertoli cells compared to the MNZ-treated mice (p<0.01), while it was significantly lower than those of the control (p<0.01) and vehicle (p<0.05) groups (Figure 6(c)).

### 3.7. Qualitative Evaluation of the Testis Histopathology

**Figure 7:**
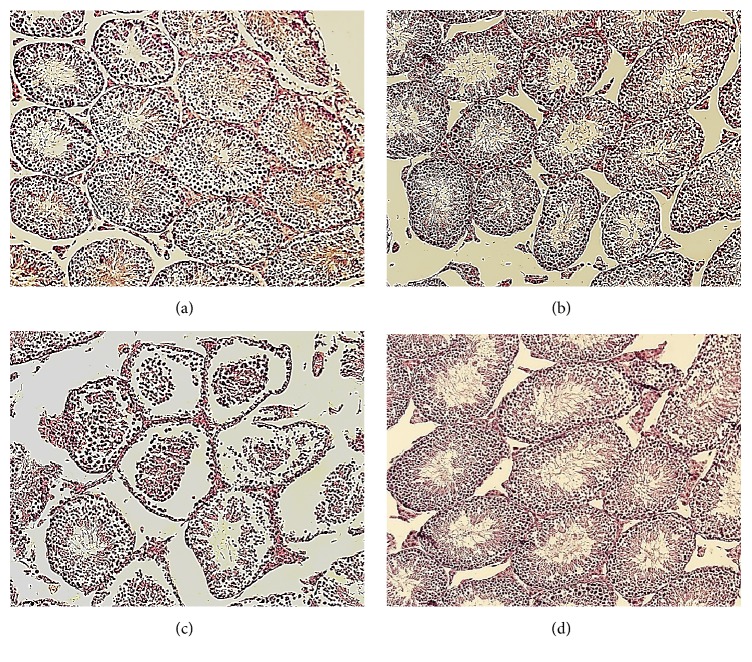
Testicular sections of adult male offspring in the different groups: (a) control group, (b) vehicle group, (c) mancozeb-treated group, and (d) vitamin E + mancozeb-treated group (H&E staining, magnification: ×200).

As illustrated in Figure 7, normal size and appearance were qualitatively observed in the structure of well-organized seminiferous tubules in the control and vehicle groups. These tubules were covered with regular rows of spermatogenic cells at different stages of differentiation and their lumens were occupied by mature spermatozoa. In contrast, the degenerative changes in the majority of the seminiferous tubules were detected in the MZN group. These histopathological changes were characterized by shrunken, disorganized seminiferous tubules with irregular appearance and incomplete spermatogenesis. The seminiferous tubules lacked spermatid and detached spermatozoa (appearing the break-off of cohorts in spermatocytes from the seminiferous epithelium). In addition, an increase occurred in the lumen diameter of the seminiferous tubules. Seminiferous tubules in the vit.E+MNZ group indicated a well-organized germinal epithelium with normal size and appearance, compared to the mancozeb-treated group, as well as an active spermatogenesis with mature sperms in the lumen.

## 4. Discussion

The application of pesticides such as mancozeb acts as a double-edge blade. The use of pesticides seems to be essential for confronting against the plant pests while it can create some undesirable side effects, especially in human. In other words, neither can be waive, nor ignore the utilization risks of them. Identifying the side effects of mancozeb usage and finding a way for preventing, repairing, and remedying the mancozeb-induced damage of developing organs can be regarded as the objectives of the applied studies in this field. Therefore, the present study aimed to investigate the effect of mancozeb on the first-generation pup testicular structure following its administration in pregnancy and lactating periods and possible protective effects of administering vitamin E.

Based on the results, the damage of testis structure in the first-generation pups was highly considerable, due to mancozeb toxification. Such damage was made by the treatment of animals by one-tenth of mancozeb lethal dose (500mg/kg) [3], which may be more than the actual mancozeb contamination of the crops and drinking water in the nature [26]. Hence, based on the results of the present and previous studies, the contamination of low-dose mancozeb in agricultural products and drinking water should not be considerably harmful. Thus, the detrimental effect of mancozeb is dose-dependent. In other words, the severity of the mancozeb impacts depends on the consumption dose, chronic or acute use, exposure timing (e.g., during sexual development in utero, childhood, and adulthood), as well as the animal model and susceptibility of the life stage [27–29]. Therefore, the harmfulness of low dose in mancozeb should be considered since developmental period and infancy are the most sensitive stage of life.

Almost all testicular measurements that were negatively impacted by mancozeb were lessened by coexposure to vitamin E. It is worth noting that the mancozeb exposure of first-generation animals in pregnancy and lactation period resulted in creating some dramatic changes in sperm parameters and testis structure such as reducing sperm parameters including number, viability, motility, and morphology, decreasing testis parameters including weight, GSI, germinal epithelium thickness, and Johnsen's score [24], along with the depletion of germ cells. Interestingly, administering vitamin E reversed these injuries considerably. Although the present study is regarded as the first report of mancozeb deleterious effects on first-generation's male reproductive system, there are some reports in the literature showing deleterious effects of mancozeb on adult male reproductive system. For example, Khan (1996) studied the effects of mancozeb exposure on murine sperm morphology and sperm count. In addition, Krishanthe (2014) evaluated the function and motility of bovine spermatozoa following short-term mancozeb exposure, the results of which confirm those in the present study [30, 31]. Comparing the results of the most recent in vitro studies with the present in vivo study while revealing the extent of the toxic effect of mancozeb on a wide range of specific cells such as Sertoli, Leydig, spermatogenic cells, and granulosa cells shows other different aspects of the damage caused by mancozeb, including the ultrastructural and morphometric alterations in the male and female reproductive organs, endocrine disruption, and mancozeb-dose-dependent-alterations [6, 28, 32, 33].

In general, the number of evidences confirming the negative link between the mancozeb and the reproductive system is increasing [1]. Further, the teratogenic impact of mancozeb was reported by considering the ability of mancozeb to cross the rat placental barrier following the administration of 2-3 g/kg mancozeb in Castro's work and 50-500 mg/kg in Rossi's work [17, 34]. Furthermore, a significant decrease in Johnsen's score and spermatogenic cells of first-generation pups in mancozeb-treated group could lead to infertility or a considerable decrease in fertility. More impairment of the fertility potential is expected when these events are synchronized with the significant decrement related to the Sertoli cells which were emphasized in the present study. However, further studies should be conducted to understand the profound and long-time effects of mancozeb on the fertility potential of first-generation pups.

The deleterious impact of mancozeb is related to hormonal imbalance and an increase in the level of reactive oxygen species (ROS) [35], which are broadly linked to phospholipid bilayer membrane [36] and disrupted cell membrane. In addition, the created oxidative stress (OS) could affect prenatal development [37]. Further, the low amount of ROS produced by sperms [38] positively influenced sperm capacitation and maturation, although its high levels could threaten fecundity. Continuous production of ROS is swept by endogenous antioxidants [39]. Breaking the balance between producing and sweeping ROS by exogenous substance such as mancozeb could lead to DNA and cell membrane damage which result in activating p53, arresting cell proliferation, and apoptosis [40]. Apoptotic and necrotic changes due to mancozeb-induced oxidative stress have already been reported [11, 41]. Such changes in the reproductive system have been reported to lessen following antioxidant administration. Therefore, male reproductive system may become at risk, and some destructive impacts of mancozeb may occur if the ROS production level is more than the oxidant defense capacity of sperm cell lineage. Antioxidants with free radical-scavenging capacity could reduce ROS with the help to aforementioned protective system and reduce the severity of damage. Vitamins such as vitamin E are powerful antioxidants which inhibits lipid peroxidation reaction of cell membranes and protecting them from the damage caused by free radicals [42]. Furthermore, vitamin E is used as a supplementation for improving human fertility and is well-known as an anti-infertility substance [43–47]. Considering the fact that vitamin E is a liposoluble vitamin and is removed slowly from the body, it may have long-term effects on the body organs. Therefore, vitamin E is almost simultaneously reached to the blood and tissues, assuming that it is given prior to the administration of mancozeb. In brief, the mechanisms by which vitamin E protects cells from oxidative stress include maintaining the natural level of glutathione as an intracellular scavenger of the free radicals, protecting cell membranes (from injuries caused by pesticides) by inhibiting peroxidation, clearing cells from ROS [16, 48], and reducing apoptosis [49, 50]. An improvement in sperm parameters which has been reported following vitamin E intake includes an increase in the quality and quantity of sperm [51] and the percentage of the sperm survival [52], as well as the maintaining the integrity of sperm's DNA from oxidative damage [53]. On the other hand, vitamin E deficiency could negatively affect reproductive performance by testicular tissue damage, testosterone synthesis reduction, and consequently a decrease of spermatogenesis process [54].

Reducing sperm parameters is considered as another reason for confirming the harmful effect of mancozeb on testis, recovering this damage significantly by vitamin E, and confirming the OS impact induced by mancozeb. The results are in line with those of Yue et al. 2010, which described the protecting effect of vitamin E on the sperm parameters by improving cell membrane and mitochondria. Since vitamin E is liposoluble and can pass through the placenta and blood-milk barrier [55, 56], it may be effective during pregnancy and lactation periods and neutralize the toxic effects of mancozeb before and after birth. These periods are the most sensitive times for developing body organs like testis. Based on some reports, testis structure and its germ cells are susceptible to the harmful substances like mancozeb. In the present study, a significant reduction occurred in the germinal and spermatid cells of first-generation seminiferous tubules, which revealed the destructive impact of mancozeb on testis structure. A reduction in spermatogenic cells of testes and serum level of testosterone has been reported after mancozeb administration [4]. However, intrauterine and after-birth impacts of mancozeb have not been evaluated yet. The results indicated that spermatogonial cells, spermatocytes, and spermatids are significantly damaged by mancozeb, and vitamin E can prevent these destructive changes via its antioxidant ability. Finally, the occurrence of testicular impairment, along with decreasing sperm parameters, and possible infertility are considered as some reasons for caution in mancozeb exposures and possible use of vitamin E in the different workers which may encounter with mancozeb exposure.

## 5. Conclusion

Based on the results, the testicular tissue in the first-generation mice exposed to mancozeb during pregnancy and lactation periods is vulnerable and may be damaged. Thus, it is recommended that labor pregnant mothers should protect themselves from exposure to mancozeb, even short- and low-dose exposures, and consume vitamin E under their physician recommendation.

## Data Availability

The data used to support the findings of this study are available from the corresponding author upon request.
